# Infusion of N‐acetyl cysteine during hypoglycaemia in humans does not preserve the counterregulatory response to subsequent hypoglycaemia

**DOI:** 10.1002/edm2.144

**Published:** 2020-05-15

**Authors:** Amir Moheet, Anjali Kumar, Yuan Zhang, Lynn Eberly, Lisa D. Coles, Elizabeth R. Seaquist

**Affiliations:** ^1^ Division of Diabetes, Endocrinology and Metabolism Department of Medicine University of Minnesota Minneapolis MN USA; ^2^ Division of Biostatistics School of Public Health University of Minnesota Minneapolis MN USA; ^3^ Department of Experimental and Clinical Pharmacology College of Pharmacy University of Minnesota Minneapolis MN USA

**Keywords:** impaired awareness of hypoglycaemia, N‐acetyl cysteine, type 1 diabetes

## Abstract

**Aim:**

Administration of N‐acetyl cysteine (NAC) during hypoglycaemia will preserve the counterregulatory response to subsequent hypoglycaemia in healthy humans.

**Methods:**

This was a randomized double‐blind cross over study where humans were given either a 60‐minute infusion of NAC (150 mg/kg) followed by a 4‐hour infusion of NAC (50 mg/kg) or saline starting 30 minutes before the initiation of a 2‐hour hypoglycaemic (HG) clamp at 8 am. After rest at euglycaemia for ~2 hours, subjects were exposed to a 2nd HG clamp at 2 pm and discharged home in euglycaemia. They returned the following day for a 3rd HG clamp at 8 am.

**Results:**

Twenty‐two subjects were enrolled. Eighteen subjects completed the entire protocol. The epinephrine response during clamp 3 (171 ± 247 pg/mL) following clamp 1 NAC infusion was lower than the response during the clamp 1 NAC infusion (538 ± 392 pg/mL) (clamp 3 to clamp 1 NAC: *P* = .0013). The symptom response during clamp 3 (7 ± 5) following clamp 1 NAC infusion was lower than the response during the clamp 1 NAC infusion (16 ± 10) (clamp 3 to clamp 1 NAC: *P* = .0003). Nine subjects experienced rash, pruritus or nausea during NAC infusion.

**Conclusion:**

We found no difference in the hormone and symptom response to experimental hypoglycaemia measured in subjects who were administered NAC as opposed to saline the day before. This observation suggests that further development of NAC as a therapy for impaired awareness of hypoglycaemia in patients with diabetes may be unwarranted.

## Graphical abstract

Infusion of the antioxidant N‐acetyl cysteine during hypoglycaemia in healthy humans did not preserve the counterregulatory response to hypoglycaemia the next day. This observation suggests that further development of NAC as a therapy for impaired awareness of hypoglycaemia in patients with diabetes may be unwarranted.

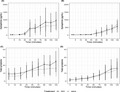

**Abbreviations**
NAC: N‐acetyl cysteine

## 1 INTRODUCTION

Hypoglycaemia occurs frequently in the lives of patients with type 1 diabetes. Recurrent hypoglycaemia episodes closely spaced in time leads to impaired awareness of hypoglycaemia in which the counterregulatory response to hypoglycaemia is markedly reduced, and the first sign of a low blood sugar is neuroglycopenia.^1^ Patients with impaired awareness of hypoglycaemia have a fourfold increased risk of experiencing severe hypoglycaemia,^2^ which by definition is an event that requires the assistance of another person to treat. The fear of experiencing hypoglycaemia and of developing impaired awareness of hypoglycaemia is the primary barrier to achieving the level of glucose control necessary to prevent the microvascular complications of the disease and contributes to the mortality seen in patients with type 1 diabetes.

To address this critical issue in diabetes management, researchers have focused on developing ways to prevent and treat impaired awareness of hypoglycaemia. Initial efforts sought to prevent hypoglycaemia from ever occurring, with the thought that in the absence of recurrent hypoglycaemia the counterregulatory response would remain sufficiently robust to warn subjects of impending hypoglycaemia whenever a rare event occurred. Cranston et al^2^ and Dagago Jack et al^3^ both demonstrated that meticulous attention to matching insulin doses to metabolic requirements over a 3 weeks period restored the counterregulatory response in well‐controlled patients with type 1 diabetes, but such an approach has been very difficult to implement in clinical practice. More recently, investigators have used information obtained in experimental studies designed to identify the pathways that link the detection of a single episode of hypoglycaemia to the blunting of the counterregulatory response during hypoglycaemia experienced in subsequent days. Such investigation has revealed the importance of opioid signalling during the first episode of hypoglycaemia and has demonstrated that blockade of opioid signalling using an intravenous administration of a drug during this first episode will preserve the counterregulatory response to hypoglycaemia the subsequent day.^4^ However, acute oral administration of the opioid antagonist naltrexone did not have an impact on the counterregulatory responses in subjects with well‐controlled type 1 diabetes^5^ and 4 weeks of oral naltrexone therapy did not restore the counterregulatory response in patients with type 1 diabetes and impaired awareness of hypoglycaemia.^6^ Future work will need to determine if the failure of naltrexone to restore the response was due to inadequate dosing or selection of patients who may be refractory to such restoration.

Reactive oxygen species generated in the brain of animals exposed to hypoglycaemia have also been linked to the blunting of the counterregulatory response to hypoglycaemia the following day. In a rodent model, administration of N‐acetyl cysteine (NAC) during episodes of hypoglycaemia prevented the blunting of the counterregulatory response, possibly by preventing the hypoglycaemia induced rise in hypothalamic reactive oxygen species known to occur during acute episodes of hypoglycaemia.^7^, ^8^ Whether NAC will be beneficial in humans is unknown. NAC is a therapy that has long been FDA approved for the treatment of acetaminophen overdose,^9^ is available as a nutritional supplement and has been used to treat a variety of human conditions including pulmonary,^10^ renal^11^ and psychiatric maladies.^12^

The purpose of this study was to test the hypothesis that NAC will be effective in the prevention and treatment of impaired awareness of hypoglycaemia. In this investigation, we performed a proof of principle experiment to determine if the intravenous administration of NAC during experimental hypoglycaemia at the maximal dose approved for use in humans prevents the expected blunting in symptom scores and epinephrine secretion during subsequent hypoglycaemia in healthy volunteers using a standard 2‐day protocol to induce impaired awareness of hypoglycaemia.^13^ We also measured plasma and red blood cell levels of NAC during the infusion period to characterize NAC's pharmacokinetics.

## 2 MATERIALS AND METHODS

Study participants were recruited from the University of Minnesota community. The protocol was approved by the Institutional Review Board. Recruitment began in 2014, and the study was completed in 2018. Inclusion criteria were age 18‐65 years, healthy status and willingness to participate in the protocol. Exclusion criteria were history of diabetes, stroke, seizures, arrhythmias, active cardiac disease; pregnancy or plan to become pregnant during the study; diagnosis of asthma (increases risk of hypersensitivity reactions to NAC), and use of antioxidants or drugs that can alter glucose metabolism.

**FIGURE 1:**
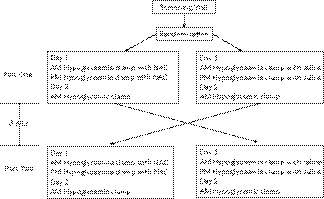
Study protocol

The study was done in the Clinical Research Unit at the University of Minnesota Medical Center. The design was a double‐blind cross over study where subjects received either NAC or saline in random order while they underwent the first of three hyperinsulinaemic hypoglycaemia clamp studies. The study protocol is shown in Figure 1. The NAC and saline experiments were separated by a minimum of 8 weeks. Subjects arrived in the fasting state at 7 am. After IV catheters were placed, subjects remained at rest for 30 minutes before being given a bolus injection of 25 mg diphenhydramine at time zero. This was immediately followed by a 60‐minute infusion of NAC (150 mg/kg) or a similar volume of saline. At minute 60, a second continuous infusion of NAC (50 mg/kg) or similar volume of saline was started and infused for 4 hours. At the discretion of the investigator, additional doses of diphenhydramine were given to subjects who developed rash or pruritus. In addition, ondansetron (4 mg) was administered intravenously to subjects who developed nausea.

At minute 30, a continuous infusion of insulin at a rate of 2.0 mU/kg/min was started and blood glucose was allowed to fall to 50 mg/dL. Insulin was given at this rate for 120 minutes during which blood glucose was clamped at 50 mg/dL using a variable infusion of glucose. Subjects were then brought to euglycaemia. At 4 hours, the continuous infusion of insulin was again started at a rate of 2.0 mU/kg/min for 120 minutes. Blood glucose was again allowed to fall to 50 mg and clamped there by a variable infusion of glucose. At 4 hours 120 minutes, subjects were brought to euglycaemia. Subjects were fed a meal and sent home. They returned the following morning and underwent a final 2‐hour hypoglycaemia step clamp with nominal targets of 75, 65, 55 and 45 mg/dL.

During each clamp study, blood glucose was measured every 5 minutes. During the first and third clamp studies, blood samples were also collected every 15 minutes for the later measurement of epinephrine and norepinephrine. Plasma and red blood cell samples were also collected every 15 minutes during day 1 for later measurement of NAC concentrations. During clamps one and three, subjects were asked to rate their hypoglycaemic symptoms at baseline and every 15 minutes using a standardized questionnaire.^14^ Part Two of the study was identical except the other treatment was given (saline was given if NAC was given during Part One; NAC was given if saline was given during Part One).

Plasma glucose concentration was measured in duplicate during the clamps using an Analox machine (Analox Instruments). Counterregulatory hormones were assayed at the Vanderbilt Diabetes Research and Training Center core laboratory. Plasma epinephrine and norepinephrine were measured by high‐performance liquid chromatography (Dionex, formerly ESA, Inc).

N‐acetyl cysteine was measured using validated high‐performance liquid chromatography coupled with triple quadrupoles mass spectrometry (HPLC‐MS/MS). Six calibration standards (range of 12.5‐1000 µg/mL NAC) and three quality control (QC) standards (low, mid, high) were prepared using diluted plasma (1:1 with 20 mmol/L ammonium formate buffer). The internal standard was 120 µg/mL deuterated NAC. All standards were analysed in triplicate or quadruplicate. Samples were incubated in a water bath for 30 minutes at 37°C and 85 rpm. Following incubation, 2 mL of methanol was added to each tube to precipitate the protein. All samples were centrifuged at 2000 rpm for 10 minutes. The organic layer was conserved and evaporated at 37°C by gaseous nitrogen (15 psi) using a TurboVap^®^ LV Concentration Evaporator. Sample contents were reconstituted in 300 μL of buffer and filtered through Acrodisc^®^ nylon syringe filters into HPLC vials for analysis. Plasma samples were analysed by a Hewlett‐Packard 1100 series (Agilent Technologies) high‐performance liquid chromatographer (HPLC) with a triple quadrupole mass spectrometer. Analytes were separated using a Zorbax Eclipse (Agilent Technologies) XDB C18 column (150 × 3.0 mm, 3.5 µ particle size) with a mobile phase consisting of 20 mmol/L ammonium formate buffer (pH 3.5) and acetonitrile (Sigma; 98:2 vol/vol). N‐acetyl cysteine was detected in negative ion mode with quantitation ions at m/z 162. The deuterated form of NAC was used as internal standard and quantified at m/z 165. The flow rate was 0.35 mL/min, and the run time was 10 minutes.

The primary outcome was defined as the mean epinephrine recorded during minutes 60‐75 of hypoglycaemia clamp 3; this is referred to below as the ‘epinephrine response’ to hypoglycaemia. A similar outcome was defined for total symptoms. If NAC prevents impaired awareness of hypoglycaemia, then the epinephrine response during the third hypoglycaemic clamp after NAC infusion will still be high, while the epinephrine response during the third hypoglycaemic camp after saline infusion will be blunted. Therefore, this within‐person NAC minus saline difference in epinephrine response will be large and positive. Assuming a one‐sided type I error of 5% and a power of 85%, this study was powered to detect a positive epinephrine response difference between NAC and saline of at least 154 units.

Data are presented as means ± standard deviations. Epinephrine and symptom responses were compared within person: clamp 1 to clamp 3 during each treatment (NAC and saline), clamp 1 NAC to clamp 1 saline and clamp 3 NAC to clamp 3 saline. A linear mixed model with effects for NAC vs. saline, order of treatment (NAC first vs. saline first), and study (Part One vs. Part Two) was used to test the primary outcome of epinephrine response during clamp 3 after NAC vs. clamp 3 after saline; a similar model was used for the secondary outcome of symptom response. Students paired t tests were used for all comparisons of clamp 1 to clamp 1 and of clamp 1 to clamp 3. We expected the clamp 1 to clamp 3 NAC comparison to be null (NAC would prevent blunting); the clamp 1 to clamp 3 saline comparison to be non‐null (saline would not prevent blunting); the clamp 1 NAC to clamp 1 saline comparison to be null (no acute infusion effect); and the clamp 3 NAC to clamp 3 saline comparison to be non‐null, specifically a greater clamp 3 response following NAC than following saline.

The impact of NAC on free epinephrine levels in plasma was evaluated in plasma samples collected during euglycaemia and hypoglycaemia to determine if NAC has the potential to release protein‐bound epinephrine and norepinephrine, causing the increase in unbound concentrations. Blood was collected from two subjects during their saline experiment's periods of euglycaemia and hypoglycaemia; plasma was separated via centrifugation. Plasma samples (n = 3‐6 per condition) were spiked with 0, 10, 100 and 1000 μg/mL NAC, and epinephrine was measured as described above.

## 3 RESULTS

Twenty‐two subjects were enrolled in the study. Two subjects developed problems with venous access during Part One of the study and were withdrawn. Eighteen subjects completed the entire protocol. One subject completed Part One having received NAC infusion, and one subject completed Part One having received saline infusion; neither returned for their Part Two. These two individuals were included in the final data set, which consisted of 10 females and 10 males, with mean age of 37.7 ± 13.7 years, mean BMI of 26.1 ± 4.2 kg/m^2^ and a mean HbA1c of 5.3 ± 0.3%.

Seven subjects (3 during NAC given in Part One, 4 during NAC given in Part Two) developed a rash or symptoms of pruritus during infusion of NAC and were administered an additional dose of diphenhydramine. In each, the symptoms rapidly resolved and the study was completed successfully. Two additional subjects (1 during NAC given in Part One, 1 during NAC given in Part Two) developed nausea during the infusion of NAC and were given doses of ondansetron. In both subjects, the symptoms rapidly resolved and the study was completed successfully. Seven of these nine adverse events were classified by the attending clinician as moderate to severe, and two were classified as mild. There were no such adverse events during any saline infusions.

**FIGURE 2:**
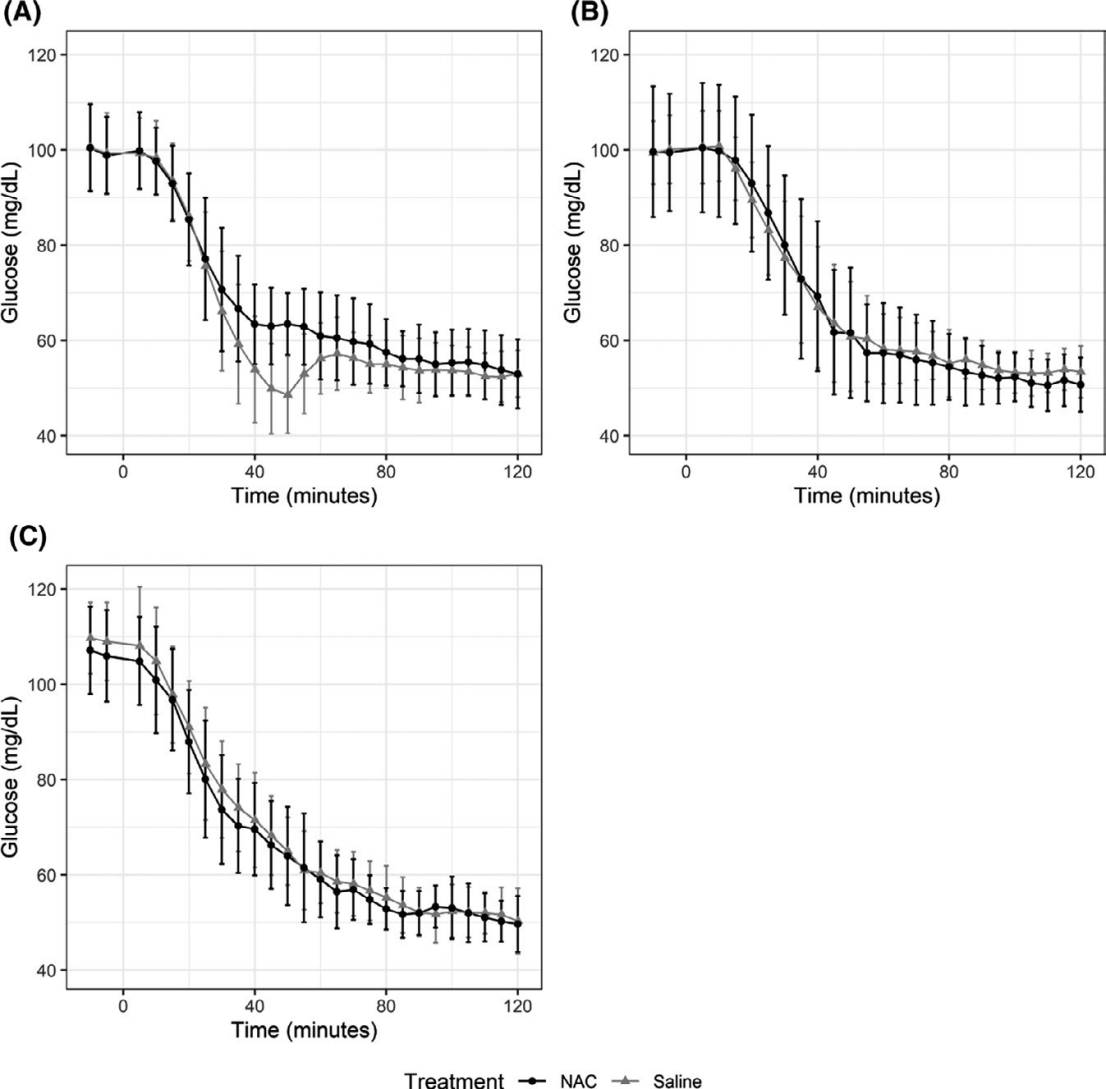
Plasma glucose concentrations achieved during clamps 1, 2 and 3. Panel A shows data collected during clamp 1, Panel B shows data collected during clamp 2, and Panel C shows data collected during clamp 3

**FIGURE 3:**
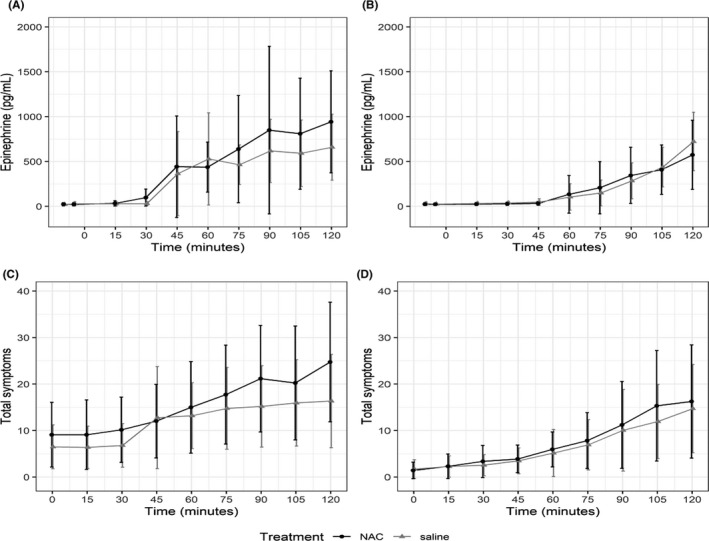
Trajectories of epinephrine and symptom responses to hypoglycaemia during clamps 1 and 3. Panels A and C shows data collected during clamp 1, Panels B and D shows data collected during clamp 3

**FIGURE 4:**
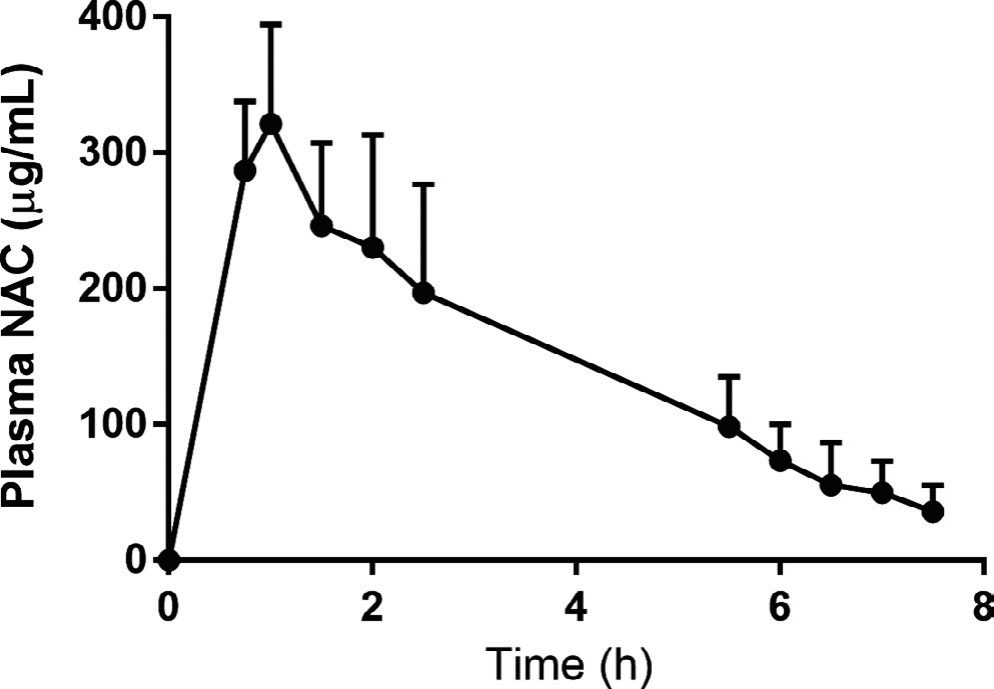
Plasma NAC levels achieved during NAC infusions during clamps 1 and 2

Hypoglycaemic targets were obtained in each of the three clamps under both treatments (Figure 2), although the glucose value dipped below 45 mg/dL before returning to 50 mg/dL during clamp one when saline was infused. During clamp one during NAC infusion, this did not occur. Epinephrine and symptoms responses are shown for each clamp and each treatment in Figure 3. The epinephrine response during the first hypoglycaemic clamp was not different during the NAC infusion (538 ± 392 pg/mL) compared to the saline infusion (496 ± 322 pg/mL) (clamp 1 NAC vs. clamp 1 saline, *P* = .57). The symptom response during the first hypoglycaemic clamp was also not different during NAC infusion (16 ± 10) compared to during saline infusion (14 ± 8 pm) (clamp 1 NAC to clamp 1 saline, *P* = .21). The mean quantity of glucose infusion per 120 minutes period of clamp 1 during the saline infusion was higher than that measured during NAC infusion (1.3 ± 1.2 vs 0.5 ± 0.7 mg/kg, *P* = .006) The NAC concentrations peaked with a mean of 321 ± 73.4 μg/mL at 60 minutes, the time of the end of the loading dose infusion, and concentrations were maintained over 100 μg/mL during the duration of the first clamp (Figure 4).

The impact of clamp 1 NAC infusion (and of clamp 1 saline infusion) on the counterregulatory response to hypoglycaemia during clamp 3 is also shown in Figure 3. Counter to our hypothesis of suppressing blunting, the epinephrine response during clamp 3 (171 ± 247 pg/mL) following clamp 1 NAC infusion was significantly lower than the response during the clamp 1 NAC infusion (538 ± 392 pg/mL) (clamp 3 to clamp 1 NAC: *P* = .0013). Similarly, the symptom response during clamp 3 (7 ± 5) following clamp 1 NAC infusion was significantly lower than the response during the clamp 1 NAC infusion (16 ± 10) (clamp 3 to clamp 1 NAC: *P* = .0003). In line with our expected induction of blunting, the epinephrine and symptom responses during clamp 3 were significantly lower than those measured during clamp 1 during the saline infusion (epinephrine: clamp 3 = 127 ± 134 pg/mL, clamp 1 = 496 ± 322 pg/mL, *P* = .0003 and symptoms: clamp 3 = 6 ± 5, clamp 1 = 14 ± 8, *P* = .0002). For our primary and secondary outcomes, no differences were found in the epinephrine (NAC: 171 ± 247; saline: 127 ± 134; one‐sided *P* = .30) and symptom (NAC: 7 ± 5; saline: 6 ± 5; one‐sided *P* = .34) responses collected during clamp 3 between those studies done after NAC or saline infusion on day one. The mean quantity of glucose infused per 120 minutes of clamp 3 was higher than that infused during clamp 1 for both NAC and saline conditions (NAC condition, clamp 1 = 0.5 ± 0.7 vs clamp 3 = 2.4 ± 1.7 mg/kg, *P* = .001; saline condition, clamp 1 = 1.3 ± 1.2 vs clamp 3 = 2.4 ± 1.5 mg/kg, *P* < .001).

## 4 DISCUSSION

In this proof of principle study, we found no difference in the counterregulatory hormone and symptom response to experimental hypoglycaemia measured in subjects who were administered NAC as opposed to saline the day before. This observation suggests that further development of NAC as a therapy for impaired awareness of hypoglycaemia in patients with diabetes may be unwarranted, particularly because we found adverse events to be more frequent in our study than in the package insert despite pretreatment with diphenhydramine.

Previous investigation in an animal model demonstrated that administration of NAC during episodes of hypoglycaemia prevents the blunting of the counterregulatory response on a subsequent day, possibly by preventing the hypoglycaemia induced rise in hypothalamic reactive oxygen species known to occur during acute episodes of hypoglycaemia.^7^ This observation, coupled with findings that NAC likely crosses the blood‐brain barrier in humans, provided the rationale for this study.^15^ However, when a similar approach was used in an animal model of diabetes, NAC administration did not preserve the counterregulatory response, perhaps because the hyperglycaemia associated with diabetes caused so much oxidative stress that NAC administration could not overcome the effect of hyperglycaemia^16^ or that pathophysiology in the previous rat model does not translate to the diabetic condition. Zhou et al suggest that dual inhibition of the S‐nitrosylation and the thioredoxin‐1 pathways in the hypothalamus might be necessary to preserve the counterregulatory response in subjects with diabetes,^16^ but our observations suggest such an approach might not work in humans.

Strengths of our study include a sufficiently large sample size to have confidence in our findings and evidence that the drug levels of NAC achieved were comparable with those associated with the treatment of acetaminophen overdose.^17^ We also were able to induce blunting of the counterregulatory response to hypoglycaemia following exposure to recurrent hypoglycaemia using our protocol, an accomplishment that has been inconsistently noted in the literature.^18^ One limitation was we performed our studies in normal controls rather than subjects with type 1 diabetes who experience the clinical syndrome of impaired awareness of hypoglycaemia. There is a growing appreciation that the exposure to recurrent hypo‐ and hyperglycaemia may be important in the pathogenesis of impaired awareness of hypoglycaemia in patients with diabetes.^19^ Another limitation is that not all subjects remained at the target range of hypoglycaemia during the final 15 minutes of clamp 3, which necessitated data analysis using samples collected during minutes 60‐75 of clamps one and three when all were maintained at target. Lucidi and colleagues have also observed such an increase in insulin resistance during an afternoon hyperinsulinaemic euglycaemic clamp that was performed after morning exposure to 3 hours of hyperinsulinaemia and hypoglycaemia.^20^

In conclusion, NAC infusion during two episodes of experimental hypoglycaemia was without effect on the counterregulatory symptoms and hormone responses to a third episode of hypoglycaemia in healthy controls. This observation suggests this currently available antioxidant is unlikely to be an effective therapy to prevent or treat impaired awareness of hypoglycaemia in diabetes.

## CONFLICT OF INTEREST

ERS has served as a consultant to Lilly, Sanofi, MannKind and Zucara.

## AUTHOR CONTRIBUTIONS

AM, AK, LDC and ERS all contributed to the study design, acquisition of data, data analysis, data interpretation, drafting/revising the manuscript and provided approval of the final version of the manuscript. LE contributed to the study design, analysis and interpretation of the data, drafting/revising the manuscript and provided approval of the final version of the manuscript. YZ contributed to the analysis and interpretation of the data, drafting/revising the manuscript and provided approval of the final version of the manuscript.

## Data Availability

Data are available upon request of authors.
